# Cost-effectiveness of digital surveillance clinics with optical coherence tomography versus hospital eye service follow-up for patients with screen-positive maculopathy

**DOI:** 10.1038/s41433-018-0297-7

**Published:** 2018-11-30

**Authors:** Jose Leal, Ramon Luengo-Fernandez, Irene M. Stratton, Angela Dale, Katerina Ivanova, Peter H. Scanlon

**Affiliations:** 1 Health Economics Research Centre, Nuffield Department of Population Health, University of Oxford, Oxford, UK; 2 Gloucestershire Retinal Research Group, Cheltenham General Hospital, Cheltenham, UK; 3 Nuffield Department of Clinical Neuroscience, University of Oxford, Oxford, UK; 4 School of Health and Social Care, University of Gloucestershire, Cheltenham, UK

## Abstract

**Background:**

Annually 2.7 million individuals are offered screening for diabetic retinopathy (DR) in England. Spectral-Domain Optical Coherence Tomography (SD-OCT) has the potential to relieve pressure on NHS services by correctly identifying patients who are screen positive for maculopathy on two-dimensional photography without evidence of clinically significant macular oedema (CSMO), limiting the number of referrals to hospitals. We aim to assess whether the addition of SDOCT imaging in digital surveillance clinics is a cost-effective intervention relative to hospital eye service (HES) follow-up.

**Methods:**

We used patient-level data from the Gloucestershire Diabetic Eye Screening Service linked to the local digital surveillance programme and HES between 2012 and 2015. A model was used to simulate the progression of individuals with background diabetic retinopathy (R1) and diabetic maculopathy (M1) following DR screening across the clinic pathways over 12 months.

**Results:**

Between January 2012 and December 2014, 696 people undergoing DR screening were found to have screen-positive maculopathy in at least one eye for the first time, with a total of 766 eyes identified as having R1M1. The mean annual cost of assessing and surveillance through the SD-OCT clinic pathway was £101 (95% CI: 91–139) as compared with £177 (95%CI: 164–219) under the HES pathway. Surveillance under an SD-OCT clinic generated cost savings of £76 (95% CI: 70–81) per patient.

**Conclusions:**

Our analysis shows that SD-OCT surveillance of patients diagnosed as R1M1 at DR screening is not only cost-effective but generates considerable cost savings.

## Introduction

Diabetes places a great economic burden on society owing to its high prevalence and increased health-care expenditures and lost productivity. Although the treatment of diabetes on its own is costly, its complications are the major contributors to health-care costs [1]. Among the main diabetes-related complications is diabetic retinopathy (DR), which is an important cause of blindness in the working age population in the UK [2]. It is possible to treat sight threatening DR effectively [3, 4] and cost-effectively [5] and screening using retinal photography has been shown to be cost-effective [6]. A common cause of sight-threatening retinopathy is diabetic macular oedema [7], which can be treated effectively by either macular laser treatment or anti-vascular endothelial growth factor (VEGF) injections [8]. However, treatment is only recommended for patients with clinically significant macular oedema (CSMO), with non-CSMO patients deriving little additional benefit from treatment [4].

In 2003, the NHS Diabetic Eye Screening Programme (NDESP) was introduced in England which uses annual digital photography with pupil dilation [9, 10]. Until 2015, NDESP recommended all screen-positive patients identified with mild non-proliferative DR and maculopathy (R1M1), moderate/severe non/pre-proliferative DR (R2M0, R2M1), or proliferative retinopathy (R3M0, R3M1) be referred to a hospital eye services (HES) for treatment assessment. However, given the limitations of two-dimensional digital photographic retinal screening for maculopathy, less than a quarter of referred M1 patients were found to have CSMO in need of treatment [11].

In January 2015, digital surveillance was included in the standard NDESP pathway for annual screening with a statement in the annual service specification “the provider shall: refer people with diabetes to digital surveillance clinics that, in the opinion of the Clinical Lead, need more frequent review and do not require referral to the HES. This should be done against local protocols based on best evidence and NDESP guidance, using appropriate technology. Surveillance clinics may interface with OCT assessment where this has been agreed with commissioners of hospital eye services” and a pathway overview diagram which is unchanged in current documents [12, 13]. The digital surveillance pathway standards [14] were updated in March 2018 to include a new standard (DES-PS-5) to ensure those in digital surveillance are seen within an appropriate time frame. No guidance has been given on grading criteria used within digital surveillance or on the use of OCT which is currently considered ‘optional’.

In June 2017, 2.7 million people were offered DR screening with 2.25 million uptake (82.2%) [15], with an epidemic posing an ever-growing strain on the screening programme and resources in the wider NHS. One way to relieve pressure on NHS services would be to improve the specificity of the current screening programme. Spectral domain optical coherence tomography (OCT) is an imaging technique that interprets reflected optical waves from a depth in the retina of 2–3 mm to produce three-dimensional images with the potential to correctly identify patients without evidence of any oedema in the macular area or CSMO, therefore limiting the number of referrals to hospitals and improving the specificity of the current screening programme [16].

Although SD-OCT imaging has been shown to be a useful adjunct in surveillance clinics for screen-positive diabetic maculopathy [16], questions remain about its cost-effectiveness in the clinical setting given its high implementation costs. Using detailed data from the Gloucestershire SD-OCT clinic surveillance programme, we aim to assess whether the addition of SD-OCT imaging surveillance in a community setting following digital retinal photography is a cost-effective intervention when screening for CSMO relative to hospital eye service assessment and follow-up.

## Methods

### Participants

We used patient-level data from the Gloucestershire Diabetic Eye Screening Service linked to the local digital surveillance programme and HES covering the period between 1st January 2009 and 31st December 2015. An anonymised cohort dataset of individuals with incident R1M1 in one eye as detected by GDESP between 1st January 2012 and 31st December 2014 was analysed. To ensure that only incident R1M1 cases were considered in our analysis, we excluded patients with previous history of R1M1 (that is, those individuals screened as R1M1 between January 2009 and December 2011). HES and SD-OCT clinic surveillance data were analysed up to 31st December 2015, allowing for at least a full year of follow-up for all incident R1M1 cases. These data included basic demographics, DR screening encounters, grading and referral outcomes, SDOCT clinic grading and referral outcomes, and HES grading, referral and treatment outcomes from two sources:

- Gloucestershire Diabetic Retinopathy Eye Screening Programme (GDESP)

i. A diabetes register for Gloucestershire provided by a regularly updated and collated data download from each Gloucestershire primary care General Practice.
ii. Every DR screening encounter, grade, outcome and referral recorded in detail since 1998 and GDESP operational pathways and data requirements managed in accordance with National Screening Programme specifications and standards.

- Gloucestershire Hospital Eye Service (HES)

i. The HES data consists of an electronic medical record (EMR) for every patient attending an HES appointment;
ii. Patient demographic Electronic Medical Record (EMR) is managed by an electronic interface with Gloucestershire Patient Administration System (PAS) and clinical episodes are recorded, by medical and allied health professional staff at the time of a patient encounter.

### Interventions under study

We estimated the cost-effectiveness of two pathways of surveillance for individuals with incident R1M1 grading detected in the diabetic screening programme:

- Technician led digital surveillance clinic including SD-OCT in a community setting
- Ophthalmologist led HES clinic assessment and follow-up

At the time of the analysis, the English National Screening Programme recommended annual screening for all patients with diabetes. The technician led digital surveillance pathway consisted of two field mydriatic digital photography (macular and disc fields) followed by a macular SD-OCT using a Topcon OCT 2000. For the HES clinical surveillance pathway, this consisted of a slit lamp biomicroscopy examination by an ophthalmologist and a macular SD-OCT using one of three SD-OCT machines used within the HES (Heidelberg Spectralis, Zeiss Cirrhus or the Topcon OCT 2000). Patients attending HES who did not need treatment were followed up in a similar fashion to that observed in the SD-OCT clinic surveillance programme in a community setting, with the exception that patients would continue to be assessed in HES. The comparison of the two pathways is shown in Online Appendix Figure A1.

### Grading criteria

The grading criteria used for retinopathy (R) and maculopathy (M) grades in the NHS Diabetic Eye Screening Programme are published [17] by NDESP. Online Appendix Table A1 reports the grading criteria and assessment of image quality for the SD-OCT images.

### Model structure

**Fig. 1:**
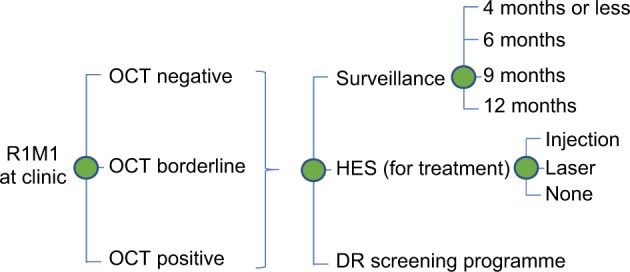
Model structure for individuals with R1M1 grading at SDOCT clinic appointment. The same model structure applies for R0M0, R1M0 and R2-R3 grading at SDOCT clinic appointment. The circles denote ‘chance’ nodes where branches meet (representing likely events) that set out the probability of an event occurring or not (e.g., OCT negative/borderline or positive given R1M1 grading at SDOCT clinic). The probabilities of events must always sum up to one at any given node. Costs were assigned to each branch including the end of the branch to value the resource use associated with each possible model pathway. These costs are combined with branch probabilities and the tree is ‘rolled back’ so that mean cost of the intervention can be estimated

A decision analytic model was developed to evaluate the impact of the two pathways of surveillance under evaluation. Given the clinic pathways and the 1 year of time horizon used in the analysis, the most appropriate model was judged to be a decision tree which was developed in Excel (Microsoft, Redmond, WA). Model structure and assumptions were informed by what was known about diabetic retinopathy, the clinic pathways of R1M1 individuals and discussions with clinical experts and statisticians involved in the project. The model was used to simulate the progression of R1M1 individuals following DR screening across the clinic pathways over 12 months (Fig. 1). Nearly all individuals who were screen positive for R1M1 were referred to SD-OCT digital surveillance clinics with a very small number referred to HES. Hence, the R1M1 population was first divided into those that attended the SD-OCT/HES clinic appointments and those that did not.

Of those attending SD-OCT clinic or HES, we further divided the R1M1 population according to the grading in the other eye at screening: R0/R1, R1M1 or R2-R3. Conditional on the grading in the other eye, we classified the R1M1 at screening according to their possible grading at SD-OCT/HES clinic appointments: R0M0, R1M0, R1M1 and R2-R3. We then further divided these subgroups according to their SD-OCT results: negative, borderline and positive. Conditional on the DR/OCT results, the individuals could be referred back to DR screening, to further surveillance (at the SD-OCT clinic or HES) or directly to HES for possible treatment of maculopathy. If referred for further surveillance, the frequency of surveillance could be: 4 months or less, 6 months, 9 months or 12 months. If referred to HES for possible maculopathy treatment, individuals might or might not receive treatment (VEGF injection or laser). Given the use of the same equipment in the digital surveillance pathway and the hospital eye services (SD-OCT) and similar levels of DR/OCT grading expertise grading in both settings, we assumed that the grading, outcomes and frequency of referral would be identical in both models.

### Costs

Following NICE recommendations, the perspective for the analysis was that of the UK NHS and the price year was 2015/16. Costs included: SD-OCT clinic surveillance appointment cost in a community setting; HES outpatient appointment costs; capital cost of SD-OCT in a hospital setting, and hospital-based treatment costs for CSMO (VEGF injection and laser) (see Online Appendix Table A2). The costs of SD-OCT surveillance clinics were obtained from a recent report evaluating community relative to hospital eye service follow-up for patients with age-related macular degeneration. [18] These authors determined the costs of monitoring review in a community settings (£51.82 in 2013/14 prices) as well as the costs of SD-OCT equipment by review (£22.99 in 2013/14 prices). Outpatient consultation costs for ophthalmology were obtained from NHS Reference Costs 2015/16. [19] We costed the first outpatient appointment following DR screening as a consultant led first appointment and the remaining appointments as consultant-led follow-up appointments. Under the HES pathway, we added the OCT equipment costs to outpatient appointment costs. The annual costs associated with laser or VEGF injections for the treatment of CSMO were obtained from patient-level data from patients attending DR screening in Gloucestershire. [20]

### Statistical analysis

We converted the Gloucestershire patient-level dataset into eye-level data so that we could follow the clinical pathway, grading and outcomes for the eye graded as R1M1 at screening over 12 months. These data were used to estimate the grade at screening in the other eye (R0-R1M0, R1M1 and R2-R3), the uptake of surveillance, the grading at community or HES surveillance clinic, and the number of surveillance appointments attended during the 12 months following initial screening. For example, the uptake rate of the surveillance clinics (community or HES-based) was determined by dividing the number of attenders, within 12 months of screening, out of all those referred at screening for HES or SD-OCT surveillance. We also used three regression models to estimate:

1. Probability of each referral outcome (i.e., referral back to DR screening, SD-OCT surveillance in a community or HES setting, HES direct referral for possible treatment of maculopathy) following the SD-OCT surveillance clinic appointment (multinomial logit);
2. Probability of the frequency of surveillance (i.e., 4 months or less, 6 months, 9 months or 12 months) following SD-OCT surveillance clinic appointment (ordered logit);
3. Probability of receiving treatment for CSMO (VEGF injection or laser).

We examined the following predictors: SD-OCT result (positive, borderline, negative), grading at surveillance clinic (R0M0, R1M0, R1M1), screening grade in other eye (R0-R1, R1M1) and other ocular conditions (e.g., macular degeneration). A predictor was deemed to be statistically significant if *p* < 0.05. Model fit was assessed using Pregibon’s link test. All analyses were performed using STATA version 14 (StataCorp, College Station, TX).

### Cost-effectiveness analysis

Following the assumption that the clinic pathways under evaluation would result in the same patient grading results, OCT results, and subsequent referrals for treatment or further surveillance, the quality of life of the R1M1 individuals was assumed to be the same. As a result, we estimated the incremental costs associated with surveillance in an SD-OCT clinic pathway relative to HES. Probabilistic sensitivity analysis (PSA) was performed where distributions were used to represent the uncertainty in the model inputs [21]. Furthermore, to capture the uncertainty and correlations between the three regression models we re-estimated the same set of models for each of the 1000 bootstraps (with replacement) of the sample dataset and saved the coefficients from the three regression models.

### Scenario analysis

We explored the impact of the scenario that once eyes were assessed at HES for the first time after diagnosis of R1M1 at screening they would no longer require further surveillance. We also explored no further surveillance for SD-OCT negative results if clinic grading was R0M0 or R1M0 in both pathways.

## Results

### Gloucester cohort study and model inputs

#### Number of eyes identified with R1M1 at screening

Between January 2012 and December 2014, 696 people with diabetes screened for diabetic retinopathy were found to be screen positive for maculopathy (M1) in at least one eye for the first time. Of these, 622 (89%) had a diagnosis of R1M1 in one eye, 4 had R1M1 in one eye and moderate to proliferative retinopathy R2M1 in the other eye (1%) and 70 (10%) had R1M1 in both eyes (Table 1), with a total of 766 eyes diagnosed as having R1M1.

**Table 1:** Baseline characteristics of patients identified with diabetic maculopathy at screening

	*n* (%) (*n* = 696)
*Gender*	
Males	440 (57)
Females	326 (43)
*Age range, years*	
<21	6 (1)
21 to 30	19 (2)
31 to 40	37 (5)
41 to 50	97 (13)
51 to 60	142 (19)
61 to 70	168 (23)
71 to 80	178 (23)
81 to 90	113 (15)
≥91	6 (1)
*Diabetes type*	
Type 1	102 (14)
Type 2	846 (86)
Duration diabetes, mean years (SD)	13 (9)
Screening grade	
*Background retinopathy and DM in one eye*	
R1M1/R0M0	181 (26)
R1M1/R1M0	441 (63)
R2M0/R1M1	2 (<1)
R2M1/R1M1	1 (<1)
R3M1/R1M1	1 (<1)
*Background retinopathy and DM in both eyes*	
R1M1/R1M1	70 (10)

#### Eye referral once identified with R1M1 at screening

**Fig. 2:**
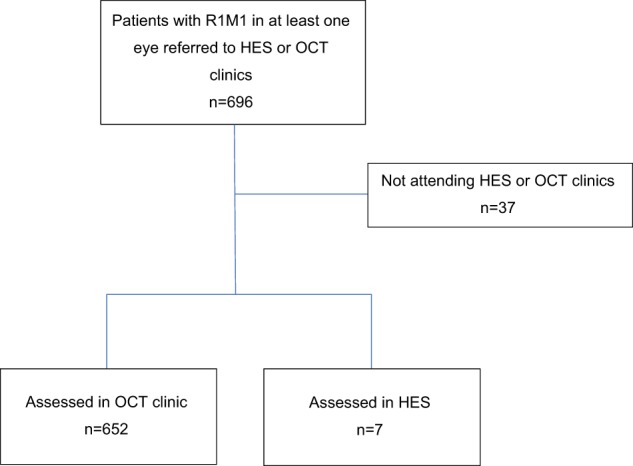
Patient pathway after diagnosis of incident background retinopathy and maculopathy at screening

All 696 individuals identified as having R1M1 at screening were referred to either HES or SD-OCT clinics, with 37 (5%) failing to attend their appointments within the first year after screening (Fig. 2). Of the remaining 659 patients, 652 (99%) were initially assessed at a digital surveillance clinic with SD-OCT after their screening episode and 7 (1%) were assessed at HES.

#### SD-OCT grading of eyes identified with R1M1 at screening

Table 2 presents the results of the grading at the SD-OCT clinic, stratified by screen grade for the other eye, for the 652 patients attending an SD-OCT clinic because of diagnosis of R1M1 at screening.

**Table 2:** Eye grading at the SDOCT clinic, stratified by screen grade of the other eye^a^

Digital surveillance grade	Screen grade of other eye R0/1M0 *n* (%)	R1M1 *n* (%)	R2/3MX *n* (%)
*R0M0*			
OCT negative	53 (10)	8 (6)	0
OCT borderline	6 (1)	0	0
OCT positive	6 (1)	0	0
*R1M0*			
OCT negative	156 (27)	37 (28)	0
OCT borderline	25 (4)	6 (5)	0
OCT positive	13 (2)	3 (2)	0
*R1M1*			
OCT negative	171 (30)	45 (35)	1 (50)
OCT borderline	77 (13)	18 (14)	0
OCT positive	54 (9)	13 (10)	1 (50)
*R2/3MX*			
OCT negative	4 (1)	0	0
OCT borderline	2 (<1)	0	0
OCT positive	1 (<1)	0	0
*RUMX*			
OCT negative	2 (<1)	0	0
OCT borderline	1 (<1)	0	0
OCT positive	1 (<1)	0	0
Total	572 (100)	130 (100)*	2 (100)

^a^Results per eye conditional on the screening grade of the other eye. MX: M0 or M1; RU: unknown retinopathy grade. For the 65 patients who were identified as having R1M1 in both eyes and had recorded SDOCT results the data has been reported for both eyes

Of the 652 patients attending SD-OCT clinic, 13 (2%) patients had missing or incomplete data (e.g., absence of an OCT result). For the remaining 639 patients, 572 (81%) were diagnosed with R1M1 at screening the other eye was diagnosed as R0/1M0 at screen (Table 2). At the first SD-OCT clinic visit, 171 (30%) of R1M1 patients at screening and with R0/1M0 in the other eye were found to be R1M1 but with an SD-OCT negative result, and 54 (9%) as R1M1 with a positive result. There were 65 patients (10%) with R1M1 in both eyes at screening (that is. 130 eyes in total). Of these patients, 2 (6%) had SD-OCT positive results in both eyes and 12 (18%) had SD-OCT positive results in one eye. Only two patients diagnosed as having R1M1 at screening were diagnosed with R2/3 in the other eye. Overall, for the 639 patients, irrespective of diabetic retinopathy grading in the SD-OCT clinic, a total of 90 (14%) patients tested SD-OCT positive and 477 (66%) tested negative.

#### Referral outcome following SD-OCT grading

For the 639 patients with recorded SD-OCT results, outcome data were missing in 11 (2%) patients. For the remaining 628 patients, 118 (19%) were returned to the DR annual screening programme, 412 (66%) continued under SD-OCT clinic surveillance, and 98 (16%) were referred to HES. Patients with SD-OCT positive results were significantly more likely to be referred to HES after controlling for SD-OCT clinic grading of the eye and the screen grading of the other eye (see Online Appendix Table A3**)**.

Of the 412 patients under continued SD-OCT clinic surveillance, 4 (<1%) were followed up in SD-OCT clinic surveillance between 1 and 3 months, 174 (42%) every 6 months, 72 (17%) every 9 months and 162 (39%) were referred to annual SD-OCT clinic surveillance. Patients with SD-OCT negative results in one eye were significantly more likely to have longer surveillance intervals than those with positive or borderline results after controlling for SD-OCT clinic grading of the eye and the screen grading of the other eye (see Online Appendix Table A4).

Including the initial SD-OCT clinic visit, the mean number of SD-OCT clinic visits for patients who were referred to SD-OCT clinic surveillance every 3, 6, 9 and 12 months was, respectively: 2.00 (S.D. 1.15); 1.95 (S.D. 0.44); 1.59 (S.D. 0.49); and 1.05 (S.D. 0.24) visits.

#### Treatment for CSMO

Of the 639 patients assessed at the SD-OCT clinic for suspected R1M1 and with SD-OCT results, 18 (3%) received treatment with laser or VEGF injection. Of these, 7 (39%) received VEGF injection and 11 (61%) received laser treatment. Results of the logistic regression (Online Appendix Table A5) showed that a SD-OCT positive result was the only significant predictor of increased likelihood of treatment (odds ratio 36, 95% CI: 10–126). Therefore, the probability of treatment given a SD-OCT positive result was 16%.

### Cost comparison between SD-OCT and HES surveillance

Online Appendix Table A2 reports the model inputs used to compare SD-OCT and HES surveillance pathways. The mean annual cost of assessing and surveillance of an eye identified as R1M1 at DR screening through the SD-OCT clinic pathway was £101 (95% CI: 91–139) per patient (Table 3). Results showed that the mean annual cost of assessing and surveillance of patient with at least one eye diagnosed as R1M1 at DR screening through the HES pathway was £177 (95% CI: 164–219) per patient.

**Table 3:** Cost-effectiveness results of SDOCT digital surveillance in a community setting vs. HES (2015/16 prices)

		Base case (£)	Scenario 1 (£)^a^	Scenario 2 (£)^b^
*SDOCT surveillance*				
Cost per patient	Mean=	101	101	99
	95% CI=	91–139	89–134	87–131
*HES pathway*				
Cost per patient	Mean=	177	142	173
	95% CI=	164–219	129–179	157–209
*SDOCT surveillance vs. HES pathway*				
Cost per patient	Mean=	−76	−41	−74
	95% CI=	−70 to −81	−37 to −46	−67 to −79

^a^Under the HES pathway, once eyes were assessed at HES for the first time after diagnosis of R1M1 at screening they would no longer require surveillance; ^b^Under both surveillance options, assuming that there was no further surveillance for SDOCT negative results if the clinic grading was R0M0 or R1M0

As a result, the mean annual cost saving per patient with one identified as R1M1 in the DR screening programme and surveilled in the SD-OCT digital surveillance pathway as opposed to a HES pathway was £76 (95% CI: 70–81), with these savings arising from less patients without maculopathy or negative SD-OCT results being referred to HES. Even if, under the HES pathway, a patient with R1M1 was assessed at HES for the first time after diagnosis of R1M1 at screening and no longer require surveillance, the SD-OCT clinic pathway still generated savings of £41 (95% CI: £37–£46) per eye assessed. Finally, assuming that there was no further surveillance for SD-OCT negative results if the clinic grading was R0M0 or R1M0 generated savings of £74 (95%CI: £67–£79) (Table 3).

## Discussion

Diabetic retinopathy screening is nationally mandated and has been shown to be cost-effective compared with no screening [22–24]. There is also a clear understanding about the appropriateness of different screening intervals for patients at differing risks of developing sight threatening DR [20]. In addition, studies have suggested that including an SD-OCT digital surveillance pathway could improve the overall efficiency of the DR screening programme [16], and its cost-effectiveness [25].

This study, based on 652 patients and over 700 eyes diagnosed as screen positive for maculopathy with background DR, and referred almost entirely for surveillance in an SD-OCT clinic, provides further evidence that surveillance of these patients in an SD-OCT clinic is not only cost-effective but generates substantial cost savings. We found that after the first surveillance visit in an SD-OCT clinic, less than 20% of patients required referral to HES as they were found not to have macular oedema or CSMO and could be safely monitored at an SD-OCT follow-up clinic or discharged back to screening if no longer screen positive for maculopathy.

At a cost of £110 and £87 for a first and follow-up face-to-face consultant visit, respectively, in ophthalmology, in addition to £24 for SD-OCT imaging, the costs of assessment at HES are considerable. By contrast, at a cost of £53 a visit including SD-OCT imaging, the costs of an SD-OCT surveillance clinic are significantly lower. By reducing the number of patients referred to HES without CSMO and referring only those who will benefit from doing so, the 1-year cost savings associated with surveillance in an SD-OCT clinic rather than HES was £76 (95% CI: 70–81), that is. a saving of 41% compared with having all these eyes assessed in HES.

Despite our study being based on a large number of eyes diagnosed as R1M1 at screening, with individual patient record linkage of data on DR screening appointments, SD-OCT clinic surveillance visits, HES appointments and eye treatments received, our study is not without limitations. First, we assumed that surveillance in an SD-OCT clinic would yield the same eye outcomes as surveillance in HES, that is we assumed that the sensitivity of SD-OCT at detecting CSMO would be equal to that of HES assessment. Previous studies and literature reviews [16, 26], suggest that SD-OCT performs very well with very similar outcomes. Second, we only established the cost implications of the two surveillance programmes over 1-year and not over the longer-term, which would most likely have resulted in larger cost savings by reviewing patients who are screen positive with R1M1 in digital surveillance clinics with SD-OCT as opposed to HES.

Finally, with no control group to compare the SD-OCT clinic surveillance programme with, we assumed that instead of attending the SD-OCT clinic for surveillance, patients attended HES directly and were then followed-up in a similar fashion in HES to that observed in the Gloucestershire SD-OCT clinic surveillance programme. We tested the implications of this assumption in our sensitivity analyses, which showed that even if eyes were assessed at HES for the first time after diagnosis of R1M1 at screening and no longer required surveillance, the SD-OCT clinic pathway still generated significant cost savings.

In conclusion, our analysis shows that SD-OCT digital surveillance of patients with diabetes, background diabetic retinopathy (R1) and evidence of diabetic maculopathy (M1) at DR screening is not only cost-effective but generates considerable cost savings.

### Summary

#### What was known before:

- The NHS Diabetic Eye Screening Programme have introduced digital surveillance clinics into their pathway for those patients who, in the opinion of the Clinical Lead, need more frequent review and do not require referral to the HES.

#### What this study adds:

- This paper is the first to show that the use of OCT in the digital surveillance pathway of the English NHS Diabetic Eye Screening Programme is both effective and cost-effective.

## Supplementary information

Supplementary material
