# Supplementary material for: Cost-effectiveness of digital surveillance clinics with optical coherence tomography versus hospital eye service follow-up for patients with screen-positive maculopathy
Source: Eye (Lond). 2018 Nov 30;33(4):640–7. doi: 10.1038/s41433-018-0297-7 (PMC6461849; doi:10.1038/s41433-018-0297-7)
Supplement: Supplementary file 1 — Supplementary material [file 41433_2018_297_MOESM1_ESM.docx]

## Supplementary Material

## Online Appendix

This online appendix provides additional methodological details to support the main manuscript (Figure **A1**, Tables **A1** and **A2**). It also provides additional results that will be of interest to the reader (Tables **A3** to **A5**)

**Figure A1. Comparison of Pathway for direct referral of people who are screen positive for R1M1 to HES and to digital surveillance**

**
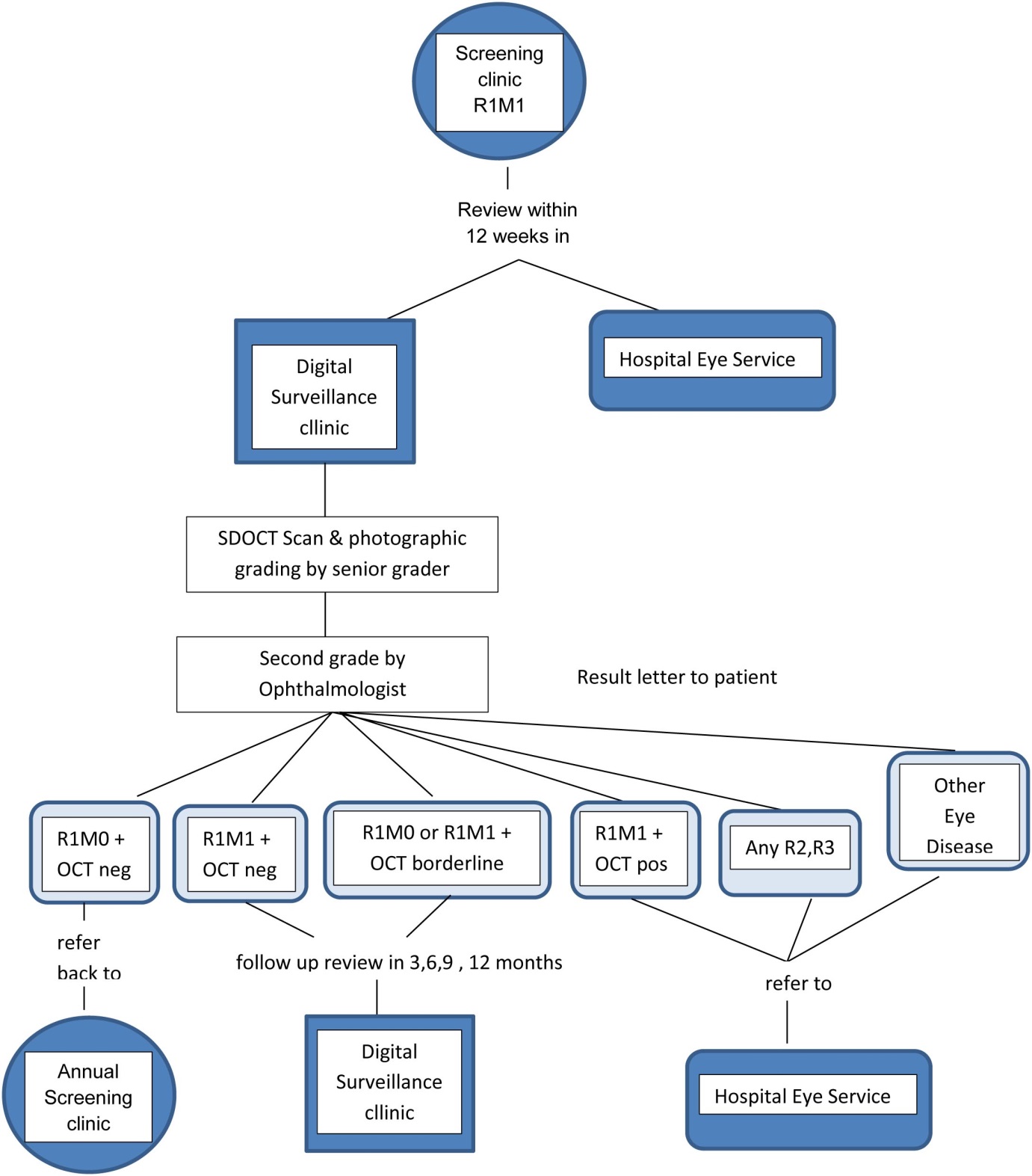
**

**Table A1**. OCT grading criteria for diabetic maculopathy in OCT surveillance

| **OCT Positive Criteria** |
| --- |
| OCT positive is defined as the following abnormalities affecting the central 1mm macular subfield: |
| - The presence of subretinal fluid, diffuse retinal thickening or parafoveal intra-retinal cysts with a change in foveal or ILM contour - Retinal thickness in the foveal central subfield ≥ 300 µm due to diabetic maculopathy - An area of retinal thickening of greater than 1 disc area the edge of which is within 1 disc diameter of the central fovea - The presence of intraretinal cystoid spaces without a change in foveal contour in the central subfield associated with a drop in VA to 6/18 or worse unless there is a clear reason why the vision is reduced e.g. dry AMD or known amblyopia. |
| **OCT Borderline Criteria**  OCT borderline is defined as:   - The presence of intraretinal cystoid spaces, or subretinal fluid, in the ‘macular area’, without any change in the ILM contour and a VA better than or equal 6/12 |
| **OCT Negative Criteria**  OCT negative is defined as:   - the absence of signs of OCT positive or borderline criteria. |
| **Other Eye Disease**  Different criteria were used in the presence of epiretinal membrane or vitreomacular traction. |
| **Adequacy of OCT Grading** |
| Adequate is defined as   - Artifacts are not present and signal strength appears optimal for interpretation of the image set.   or   - artifacts are present and/or signal strength is reduced, but there is still sufficient intensity to distinguish major interfaces across the entire dataset. |
| Inadequate is defined as   - Severe artifacts are present (e.g. “impossible” deviations in retinal contour) or the signal strength is reduced across the image set to the extent that major interfaces cannot be clearly identified. |

**Table A2**. Model inputs: Unit costs and probabilities

| **Resource use/costs (2015/16)** | **Mean** | **Distribution** | **Source** |
| --- | --- | --- | --- |
| SDOCT surveillance clinic appointment | £53 | Fixed | Reeves 2016^15^ |
| Consultant-led Ophthalmology first appointment in HES* | £134 | Fixed | NHS Reference costs 2015/16 and Reeves 2016 |
| Consultant-led Ophthalmology follow up appointment in HES* | £111 | Fixed | NHS Reference costs 2015/16 and Reeves 2016 |
| Annual cost of laser treatment | £1,134 | Fixed | Scanlon 2015 |
| Annual cost of injection treatment | £814 | Fixed | Scanlon 2015 |
| Proportion of treatment that was injection | 39% | Beta(7,11) | This study |
| Visits by SDOCT surveillance frequency |  |  |  |
| Every 3 months | 2.00 | Normal(2.00,0.58) | This study |
| Every 6 months | 1.95 | Normal(1.95,0.03) | This study |
| Every 9 months | 1.59 | Normal(1.59,0.05) | This study |
| Every 12 months | 1.05 | Normal(1.05,0.02) | This study |
| **Probabilities** |  |  |  |
| Other eye at screening |  |  |  |
| R0-R1M0 | 81% | Residual | This study |
| R1M1 | 18% | Beta(140,626) | This study |
| R2-R3 | 1% | Beta(4,762) | This study |
| Uptake rate | 95% | Beta(727,39) | This study |
| Referral outcome following SDOCT grading | Table A3 | Bootstrap | This study |
| SDOCT surveillance intervals | Table A4 |  | This study |
| Other eye R0-R1M0 at screening |  |  |  |
| R0M0 at SDOCT grading | 11% | Beta(65,503) | This study |
| R1M0 at SDOCT grading | 34% | Beta(194,374) | This study |
| R1M1 at SDOCT grading | 53% | Residual | This study |
| R2/3MX at SDOCT grading | 1% | Beta(7,561) | This study |
| Other eye R1M1 at screening |  |  |  |
| R0M0 at SDOCT grading | 6% | Beta(8,122) | This study |
| R1M0 at SDOCT grading | 35% | Beta(46,84) | This study |
| R1M1 at SDOCT grading | 58% | Residual | This study |
| R2/3MX at SDOCT grading | 0% | Fixed | This study |
| Other eye R2/3MX at screening |  |  |  |
| R0M0 at SDOCT grading | 0% | Fixed | This study |
| R1M0 at SDOCT grading | 0% | Fixed | This study |
| R1M1 at SDOCT grading | 100% | Fixed | This study |
| R2/3MX at SDOCT grading | 0% | Fixed | This study |
| Other eye R0-R1M0 at screening and R0M0 at SDCOT grading |  |  |  |
| OCT negative | 82% | Residual | This study |
| OCT borderline | 9% | Beta(6,59) | This study |
| OCT positive | 9% | Beta(6,59) | This study |
| Other eye R0-R1M0 at screening and R1M0 at SDCOT grading |  |  |  |
| OCT negative | 80% | Residual | This study |
| OCT borderline | 13% | Beta(25,169) | This study |
| OCT positive | 7% | Beta(13,181) | This study |
| Other eye R0-R1M0 at screening and R1M1 at SDCOT grading |  |  |  |
| OCT negative | 57% | Residual | This study |
| OCT borderline | 25% | Beta(77,225) | This study |
| OCT positive | 18% | Beta(54,248) | This study |
| Other eye R0-R1M0 at screening and R2/3MX at SDCOT grading |  |  |  |
| OCT negative | 57% | Residual | This study |
| OCT borderline | 29% | Beta(2,7) | This study |
| OCT positive | 14% | Fixed | This study |
| Other eye R1M1 at screening and R0M0 at SDCOT grading |  |  |  |
| OCT negative | 100% | Fixed | This study |
| OCT borderline | 0% | Fixed | This study |
| OCT positive | 0% | Fixed | This study |
| Other eye R1M1 at screening and R1M0 at SDCOT grading |  |  |  |
| OCT negative | 80% | Residual | This study |
| OCT borderline | 13% | Beta(6,40) | This study |
| OCT positive | 7% | Beta(3,43) | This study |
| Other eye R1M1 at screening and R1M1 at SDCOT grading |  |  |  |
| OCT negative | 59% | Residual | This study |
| OCT borderline | 24% | Beta(18,58) | This study |
| OCT positive | 17% | Beta(13,63) | This study |

*includes SDOCT equipment costs of £24 per appointment

**Table A3**. Multinomial regression to assess the predictors of referral outcome following SDOCT grading

|  | **Coefficient** | **SE** | **95% CI** |
| --- | --- | --- | --- |
| **Continued surveillance in an SDOCT clinic** |  | | |
| SDOCT positive | 1.07 | 1.10 | -1.09 to 3.24 |
| SDOCT grading of R0M0 | -5.91 | 1.03 | -7.93 to -3.88 |
| SDOCT grading of R1M0 | -5.34 | 1.01 | -7.32 to -3.35 |
| Screen grading of other eye of R0/1 | -1.37 | 0.41 | -2.18 to -0.56 |
| Constant | 6.90 | 1.08 | 4.79 to 9.01 |
|  | | | |
| **Refer to HES** |  |  |  |
| SDOCT positive | 5.60 | 1.09 | 3.47 to 7.74 |
| SDOCT grading of R0M0 | -6.47 | 1.22 | -8.87 to -4.07 |
| SDOCT grading of R1M0 | -5.55 | 1.07 | -7.64 to -3.45 |
| Screen grading of other eye of R0/1 | -1.99 | 0.53 | -3.03 to -0.94 |
| Constant | 4.89 | 1.12 | 2.70 to 7.08 |

**Table A4.** Ordered logistic regression to assess the predictors of SDOCT surveillance intervals

|  | **Coefficient** | **SE** | **95% CI** |
| --- | --- | --- | --- |
| SDOCT result |  |  |  |
| Positive | Reference case | | |
| Negative | 2.66 | 0.28 | 2.10 to 3.21 |
| SDOCT grading |  |  |  |
| R1M1 | Reference case | | |
| R0M0 | 2.16 | 0.48 | 1.22 to 3.11 |
| R1M0 | 1.44 | 0.25 | 0.96 to 1.93 |
|  |  |  |  |
| Screening grading of other eye |  |  |  |
| R0/1 | 0.62 | 0.24 | 0.14 to 1.10 |
| Cut between <3- and 6-month surveillance | -2.82 | 0.51 | -3.83 to -1.82 |
| Cut between 6- and 9-month surveillance | 2.66 | 0.35 | 1.98 to 3.34 |
| Cut between 9- and 12-month surveillance | 3.63 | 0.37 | 2.90 to 4.36 |

**Table A5.** Logistic regression to assess the predictors of treatment if incident R1M1 at screening

|  | **Coefficient** | **SE** | **95% CI** |
| --- | --- | --- | --- |
| SDOCT result positive | 3.57 | 0.65 | 2.30 to 4.84 |
| Constant | -5.19 | 0.58 | -6.3 to -4.06 |
